# A rapid and highly sensitive multiple detection of human adenovirus type 3, type 7 and respiratory syncytial virus by recombinase‐aided reverse transcription PCR

**DOI:** 10.1002/jcla.24889

**Published:** 2023-05-03

**Authors:** Guohao Fan, Xiaozhou He, Ruiqing Zhang, Fengyu Tian, Xiuli Sun, Mengyi Zhang, Fengyu Li, Xinxin Shen, Xuejun Ma

**Affiliations:** ^1^ National Institute for Viral Disease Control and Prevention Chinese Center for Disease Control and Prevention Beijing China; ^2^ Center for Biosafety Mega‐Science Chinese Academy of Sciences Wuhan China; ^3^ Hebei Medical University Shijiazhuang China

**Keywords:** highly sensitive, multiplex detection, respiratory viruses, RT‐qPCR, RT‐RAA

## Abstract

**Background:**

Polymerase chain reaction (PCR) has been widely used for many pathogen detection. However, PCR technology still suffers from long detection time and insufficient sensitivity. Recombinase‐aided amplification (RAA) is a powerful nucleic acid detection tool with high sensitivity and amplification efficiency, but its complex probes and inability of multiplex detection hinder the further application of this technology.

**Methods:**

In this study, we developed and validated the multiplex reverse transcription recombinase‐aided PCR (multiplex RT‐RAP) assay for human adenovirus 3 (HADV3), human adenovirus 7 (HADV7), and human respiratory syncytial virus (HRSV) within 1 h with Human RNaseP protein as a reference gene to monitor the whole process.

**Results:**

Using recombinant plasmids, the sensitivity of multiplex RT‐RAP for the detection of HADV3, HADV7, and HRSV was 18, 3, and 18 copies per reaction, respectively. The multiplex RT‐RAP showed no cross‐reactivity with other respiratory viruses, demonstrating its good specificity. A total of 252 clinical specimens were tested by multiplex RT‐RAP and the results were found to be consistent with those of corresponding RT‐qPCR assays. After testing serial dilutions of selected positive specimens, the detection sensitivity of multiplex RT‐RAP was two to eightfold higher than that of corresponding RT‐qPCR.

**Conclusion:**

We conclude the multiplex RT‐RAP is a robust, rapid, highly sensitive, and specific assay with the potential to be used in the screening of clinical samples with low viral load.

## Graphical abstract

A novel nested nucleic acid amplification assay that integrates isothermal amplification technique RAA and traditional qPCR technique. The technique starts with a first step of rapid enrichment by RAA technology, followed by a second step of PCR amplification and TaqMan probe detection. This method is a robust, rapid, highly sensitive, and specific assay with the potential to be used in the screening of clinical samples with low viral load.

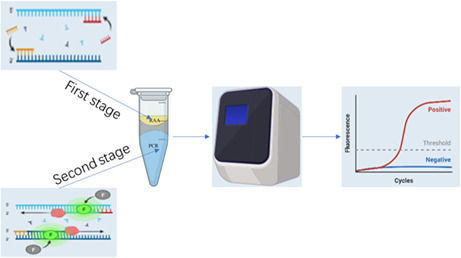

## 1 INTRODUCTION

Human adenovirus (HADV) and human respiratory syncytial virus (HRSV) are DNA and RNA viruses, respectively, of which human adenovirus types 3 and 7 (HADV3 and HADV7) are the most important pathogens causing epidemics in many countries and regions, as well as causing lower respiratory tract infections and even death.^1^, ^2^, ^3^, ^4^ Respiratory infections with HRSV can lead to bronchitis, bronchopneumonia, severe pneumonia, etc. In addition, studies have shown that HRSV is a major cause of pneumonia and bronchiolitis in infants.^5^, ^6^, ^7^ The establishment of rapid and sensitive detection methods for these viruses is of great importance for the prevention and treatment of diseases. The main diagnostic methods for infectious diseases are nucleic acid amplification technology and immunological methods, PCR as the gold standard for virus diagnosis has made a great contribution in the prevention and control of the epidemic.^8^, ^9^, ^10^ However, PCR suffers from long detection time and insufficient sensitivity.

Recently, we reported a novel two‐stage nucleic acid amplification technique recombinase‐aided PCR (RAP) by combining recombinase ‐aided amplification (RAA) with qPCR in a single closed‐tube and demonstrated that RAP revealed ultra‐high sensitivity (detection limits for HADV3 and HADV7 can reach single copy) and specificity, and could detect nucleic acids in less than 1 h, superior to current conventional qPCR assays.^11^ However, RAP has some limitations. First, RAP involves adding RAA mix to the cap of the PCR tube to complete the first stage of the RAA reaction, and then centrifuging the completed RAA product and mix with the qPCR buffer, which is cumbersome and relatively unstable. In addition, our previous study only demonstrated the effectiveness of RAP in single‐plex DNA detection, but not in RNA virus and multiplex applications. Finally, the detection of internal reference targets was not included in RAP.

To overcome the drawbacks of RAP in multiplex and RNA virus detection, in this study, we developed a multiplex reverse transcription recombinase‐aided PCR (multiplex RT‐RAP) assay by using HADV3, HADV7, and HRSV to examine the ability of RAP in multiplex detection of DNA and RNA viruses. The human‐derived Human RNaseP protein p38 gene (HRPP) was added in this method to improve the authenticity and credibility of the assay results. A paraffin (docosane) was added as a barrier to separate the RAA mix from the quantitative PCR (qPCR) mix. The multiplex RT‐RAP was further validated with clinical samples.

## 2 METHOD

### 2.1 Sample collection and nucleic acid extraction

The clinical specimens of the respiratory tract were obtained from the Hunan Provincial Center for Disease Control and the Capital Institute of Pediatrics. The types of clinical specimens contained pharyngeal swabs and alveolar lavage fluid. The total DNA and RNA from 200 μL of these clinical specimens were extracted by Viral RNA/DNA Isolation Kit (Tianlong) following the manufacturer's instructions. The nucleic acids were eluted in 50 μL of elution buffer into 1.5 mL EP tube and stored at −80°C refrigerator.

### 2.2 Primer, probe, and plasmid construction design

Complete sequences of HRSV were downloaded from the Nation Center for Biotechnology Information database (NCBI: https://www.ncbi.nlm.nih.gov/) and aligned using Vector NTI 11.5.1 to locate the highly conserved regions. RAA primers (outer primers of RAP) for HADV3, HADV7, HRSV, and HRPP were cited from previous studies in our laboratory.^4^, ^5^, ^12^ The qPCR primers (inner primers) and probes of HRSV and HRPP were designed using Primer 6 software and further evaluated using Primer‐Blast in the NCBI website. The sequences of primers and probes are presented in Table 1. All of them were synthesized and purified by Shanghai Bioengineering.

**TABLE 1:** Primer and probe sequences used in this study.

Assay	Primer/probe	Sequence (5′–3′)	Source
First‐stage RAA	HADV3 outer‐F	ATTCCGGCACAGCTTACAATTCACTCGCTCC	Wang et al. ^4^, ^5^, ^12^
	HADV3 outer‐R	TCAGTAGTGG TAATGTCTTT CCCAATTTGC	
	HADV7 outer‐F	ACAACGGGAGAAGACAATGCCACCACATACAC	
	HADV7 outer‐R	TCCATCAATATCAGTCCATGATTCTTCTCC	
	HRSV outer‐F	TCCYAATTGTATAGCATTCATAGGTGAAGGAGC	
	HRSV outer‐R	TTGCATCTGTAGCAGGAATGGTYAAATTYTCAC	
	HRPP outer‐F	GCTTAAAATGTGTTCTAGCCTTGGCGTTCA	
	HRPP outer‐R	CAGAGGTTCAGTCTCTAAATTTTCCCCAGA	
Second‐stage qPCR/RT‐qPCR	HADV3 inner‐F	AGCTTACAATTCACTCGCTCC	This study
	HADV3 inner‐R	TAGTGGTAATGTCTTTCCCA	
	HADV7 inner‐F	AGAAGACAATGCCACCACAT	
	HADV7 inner‐R	CAGTCCATGATTCTTCTCC	
	HRSV inner‐F	TTGTATAGCATTCATAGGTG	
	HRSV inner‐R	TGTAGCAGGAATGGTYAAAT	
	HRPP inner‐F	ATGTGTTCTAGCCTTGGCGTTC	
	HRPP inner‐R	AGGTTCAGTCTCTAAATTTTC	
	HADV3 P	FAM‐ACAATGCAGTAACTACCACCACAAAC‐BHQ	
	HADV7 P	HEX‐AAGACATTACTGCAGACAACAAGCC‐BHQ	
	HRSV P	Cy5‐ACAGAAGTYTGAAAGATTGC‐BHQ	
	HRPP P	ROX‐ATGGTACACTTAAACTGGGGAC‐BHQ	
qPCR/RT‐qPCR	HADV F	GGYCCYAGYTTYAARCCCTAYTC	Qi et al.^5^, ^13^
	HADV R	AAYTTGAGGYTCTGGYTGATCKG	
	HADV3 probe	HEX‐ACAATGCAGTAACTACCACCACAA‐MGB	
	HADV7 probe	Cy5‐TTACTGCAGACAACAAGCCCAT‐MGB	
	HRSV F	CACWGAAGATGCWAATCATAAATTCA	
	HRSV R	GTATYTTTATRGTGTCTTCYCTTCCTAACC	
	HRSV probe	FAM‐TAATAGGTATGTTATATGCKATGTC‐BHQ	

### 2.3 Strategy of multiple RT‐RAP assay

**FIGURE 1:**
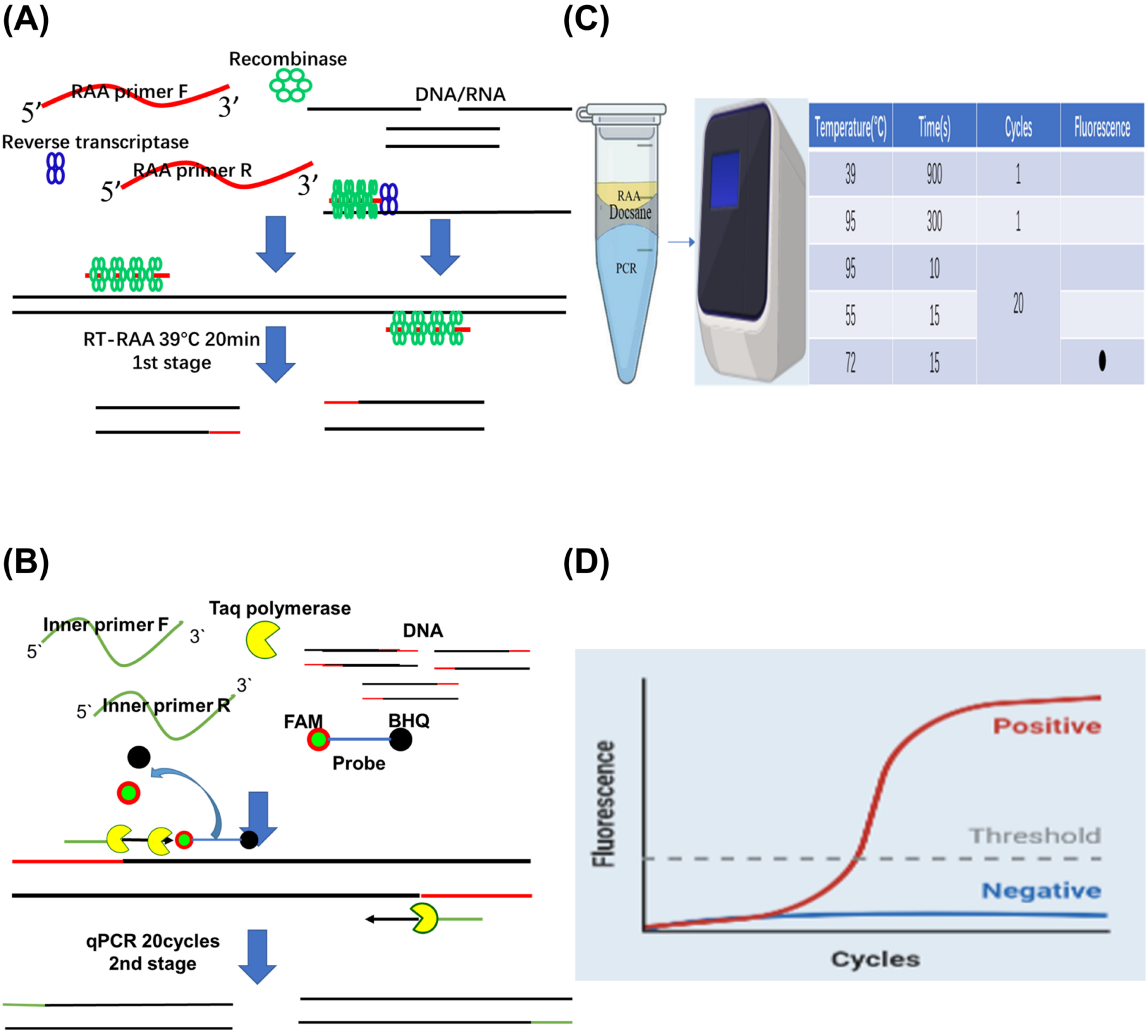
The working principle of reverse transcription recombinase‐aided PCR (RT‐RAP). (A) RT‐RAA for 20 min at 39°C (first stage of RAP). (B) qPCR 20 cycles (second stage of RAP). (C) The reaction tube comprises two compartments. The compartment above docosane contains the RAA mix and the compartment below docosane houses the qPCR mix. (D) The results can be revealed by real‐time fluorescence curves with qPCR machine.

The multiplex RT‐RAP assay consists of two stages, the first stage is the RT‐RAA reaction, which reacts at a constant temperature of 39°C, the second stage is the qPCR reaction, which undergoes three processes: denaturation, annealing, and extension (Figure 1). Since RT‐RAA mixes and PCR mixes are not compatible, physical isolation of the two mixes is required. Here, a barrier called docosane (melting point 45°C) is added to separate the two compartments. The RT‐RAA reaction is completed in the compartment above docosane. During qPCR pre‐denaturation of the second stage, docosane melts into a liquid state with less density than the RT‐RAA mixture, enabling RT‐RAA mixture mixes with the qPCR mixture in the compartment below docosane to start the second‐stage qPCR reaction, which is completed after 20 cycles. The whole multiple RT‐RAP assay lasts for 1 h.

### 2.4 Optimization of multiplex RT‐RAP assay working conditions

The optimal reaction conditions such as the volume of docosane, annealing temperature of qPCR, reaction time of RT‐RAA, and primer concentration of qPCR were explored. First, to ensure that the two mixes could be effectively isolated before the RT‐RAA reaction was completed, we explored the isolation effect of adding docosane in volumes of 15, 20, 25, and 30 μL, respectively. In addition, we explored the optimal annealing temperature for each primer at a plasmid concentration of 10^4^ copies per reaction. Next, we examined the time effect of RT‐RAA reaction at 5, 10, 15, and 20 min using HADV7 as an example with the plasmid template concentration of 1000, 100, 10, and 1 copies/reaction, respectively. Finally, we optimized and adjusted the concentration of qPCR primers in the reactions.

### 2.5 Sensitivity and specificity of multiplex RT‐RAP assay

The sensitivities of multiplex RT‐RAP were measured with the serial dilutions of the HADV3, HADV7, and HRSV plasmids ranging from 10^0^ to 10^5^ copies/μL in nuclease‐free water. Meanwhile, the specificity of this method was verified by testing previously defined other pathogens‐positive clinical samples, such as SARS‐CoV‐2, parainfluenza virus, rhinovirus, influenza virus A, influenza virus B, human metapneumovirus, human boca virus, and other HADV species‐A (HADV31), B (HADV14 and 55), C (HADV1, 2, 5, 6, and 57), and E (HADV4). The procedures were operated according to the optimized working conditions.

### 2.6 Comparing the clinical performance of qPCR and RT‐RAA versus multiplex RT‐RAP

The clinical samples were simultaneously tested by previously reported RT‐qPCR, RT‐RAA, and multiplex RT‐RAP. Subsequently, three positive clinical samples for each of HADV3, HADV7, and HRSV were selected, fourfold serially diluted and detected by RT‐qPCR, RT‐RAA, and multiplex RT‐RAP. The results were analyzed by SPSS 26 software.

## 3 RESULTS

### 3.1 Multiple RT‐RAP reaction condition optimization

**FIGURE 2:**
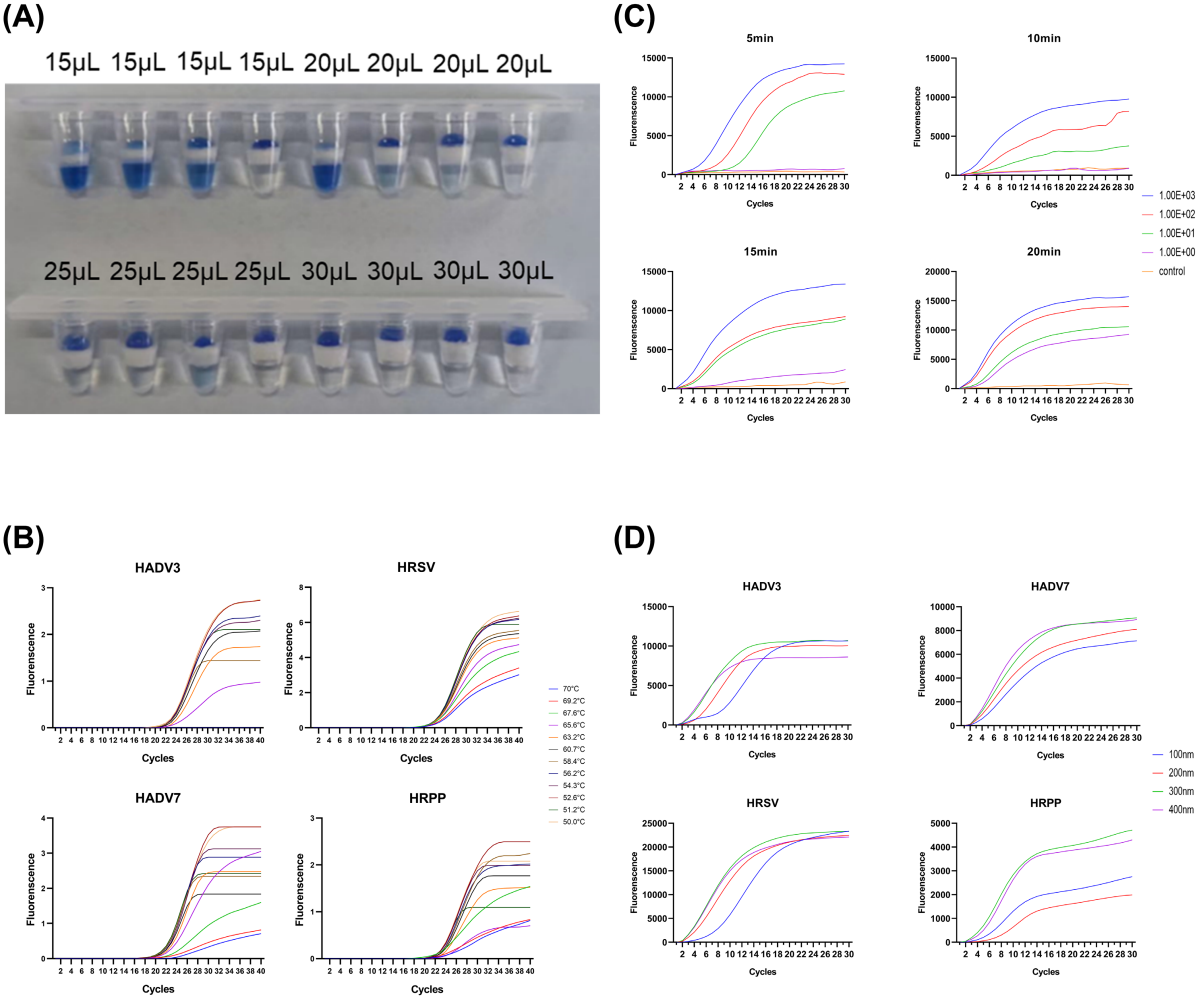
(A) The blocking effect of docosane on the isolation of RT‐RAA reaction from qPCR mix. (B) Amplification curve of HADV3, HADV7, HRSV, and HRPP at different annealing temperatures ranging from 50°C to 70°C. (C) Amplification curves of HADV3, HADV7, HRSV, and HRPP ranging from 1.00E+00 to 1.00E+03 copies/reaction at different RT‐RAA time. (D) Amplification curve of HADV3, HADV7, HRSV, and HRPP at different inner primer concentrations.

The volume ratio of RT‐RAA mix to PCR mix was previously determined to be 1:4.^11^ Our results showed that an effective barrier could be formed when 30 μL of docosane was added (Figure 2A). In addition, the annealing temperature of the PCR stage exhibited a significant influence on the amplification efficiency of the reaction. Too high annealing temperature will reduce the amplification efficiency, while too low will produce nonspecific amplification. Our experimental results showed that the best amplification efficiency was achieved when the annealing temperature was 55°C, and at the same time no obvious nonspecific amplification occurred at 55°C (Figure 2B). The time of RT‐RAA reaction was an important factor affecting the efficiency of whole multiple RT‐RAP reaction. Our results indicated that the best amplification was achieved when multiple RT‐RAA reaction time of 20 min was used (Figure 2C). To be further, we optimized the qPCR primer concentrations. The results of the study found that the final concentration of primers for RT‐RAA was 400 nM (each 50 nM) in 10 μL mix. The PCR primer concentration was finalized to 400 nM (each 50 nM) and the probe 100 nM (each 25 nM) in a final 50 μL mix (Figure 2D).

Accordingly, we finalized the optimal reaction procedure for multiplex RT‐RAP. The RT‐RAA reaction mix was prepared according to the manufacturer (Qitian, Bio‐tech Co., Ltd.). The RT‐RAA pellet was dissolved with rehydration buffer, nuclease‐free water, 140 nM of each RAA primers, 14 mM of magnesium acetate, and then the mix was divided into four RT‐RAP volume and added 1–2 μL template that were called mix A (10 μL). The pre‐qPCR mix was called as mix B that was prepared following manufacturer's protocol but reduced the volume of MgCl_2_ (Entrans qPCR Probe Set V2, ABclonal). The mix B (40 μL) contained 200 nM of each qPCR primers and 100 nM of each qPCR probe, 0.5 mM of each dNTP, qPCR buffer, and nuclease‐free water. The RT‐RAP mix consisted of mix A, the barrier (30 μL docosane), and mix B, the mix A was above the barrier and mix B was below the barrier. The procedure of RT‐RAP was as follows: 39°C for 20 min, 95°C for 5 min, followed by 20 cycles at 95°C for 10 s, 55°C for 15 s, and 72°C for 15 s. The whole detection time was within 1 h, which is faster than traditional RT‐qPCR.

### 3.2 Sensitivity and specificity of multiple RT‐RAP assay

**FIGURE 3:**
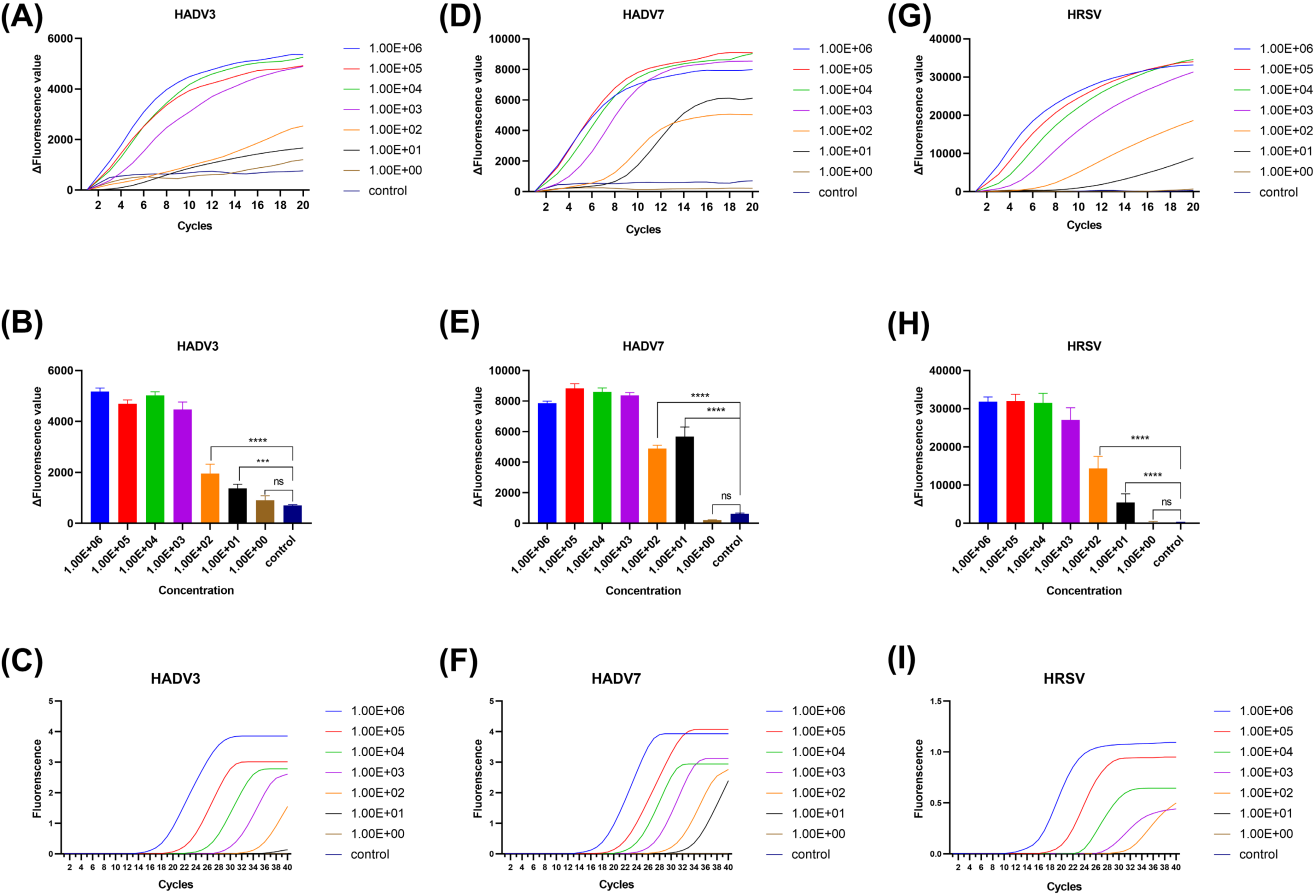
(A, D, G) Amplification curve of HADV3, HADV7, and HRSV ranging from 1.00E+00 to 1.00E+06 copies/reaction by multiplex RT‐RAP. (B, E, H) Fluorescence value of HADV3, HADV7, and HRSV ranging from 1.00E+00 to 1.00E+06 copies/reaction by multiplex RT‐RAP. (C, F, I) Amplification curve of HADV3, HADV7, and HRSV ranging from 1.00E+00 to 1.00E+06 copies/reaction by RT‐qPCR.

The sensitivity of multiplex RT‐RAP for simultaneous detection of HADV3, HADV7, and HRSV was determined by detecting serial dilutions of the plasmids. The sensitivity of the assay was determined by Probit regression analysis for HADV3, HADV7, and HRSV to be 18, 3, and 18 copies per reaction, respectively (Table 2). During the detection, no fluorescence signal was detected in any of the blank controls (Figure 3). In addition, we used SARS‐CoV‐2, parainfluenza virus, rhinovirus, influenza virus A, influenza virus B, human metapneumovirus, human boca virus, and other HADV species‐A (HADV31), B (HADV14 and 55), C (HADV1, 2, 5, 6, and 57), and E (HADV 4) positive clinical specimens to verify the specificity of the method, and no positive signal was detected, indicating no cross‐reaction with these viruses.

**TABLE 2:** Assay data used for calculating the detection limit of HADV3, HADV7, and HRSV.

Copies/reaction	No. of positive samples tested by the RAP assay for detection of HADV 3, HADV 7, and RSV		
	HADV3	HADV7	RSV
1.00E+05	8/8	8/8	8/8
1.00E+04	8/8	8/8	8/8
1.00E+03	8/8	8/8	8/8
1.00E+02	8/8	8/8	8/8
1.00E+01	6/8	8/8	6/8
1.00E+00	0/8	2/8	0/8
Control	0/8	0/8	0/8

### 3.3 Clinical performance of multiple RT‐RAP versus qPCR and RT‐RAA

Totally, 252 clinical specimens were tested simultaneously by RT‐qPCR, RT‐RAA, and multiplex RT‐RAP methods to evaluate the clinical performance of multiplex RT‐RAP assays. Multiplex RT‐RAP identified 28 HADV3 positive specimens (CT value from 15.9 to 36.0), 39 HADV7 positive specimens (CT value from 16.3 to 35.6), and 41 HRSV positive specimens (CT value from 15.2 to 31.4), which was consistent with those of RT‐qPCR/qPCR and RT‐RAA/RAA. Compared with RT‐qPCR and RT‐RAA assay, both the specificity and sensitivity of the multiplex RT‐RAP assay using 252 clinical samples were 100% (Table 3), the HRPP reference genes were all positive in the detection of the specimens, serving as a reliable monitoring of the entire reaction. Finally, we randomly selected three clinical samples positive for each of the three viruses and serially diluted these samples followed by both RT‐qPCR and multiplex RT‐RAP assays. The results showed that the sensitivity of multiplex RT‐RAP assay for HADV3 and HRSV was two to fourfold higher than that of RT‐qPCR, while the sensitivity of HADV7 was four to eightfold higher. These results demonstrated that our multiplex RT‐RAP assay has high sensitivity and specificity.

**TABLE 3:** Detection of HADV3, HADV7, and HRSV in clinical samples.

Virus	RT‐qPCR		RT‐RAP		RT‐RAA		Agreement
	Positive	Negative	Positive	Negative	Positive	Negative	
HADV3	28	224	28	224	28	224	100%
HADV7	39	213	39	213	39	213	
HRSV	41	211	41	211	41	211	

## 4 DISCUSSION

Since the outbreak of SARS‐CoV‐2, infectious diseases caused by respiratory viruses have received increasing attention, and early detection, treatment, and diagnosis are of great significance for the control, treatment, and prognosis of diseases caused by respiratory viruses.^8^, ^13^, ^14^ On the other hand, patients with respiratory viral infections have similar clinical symptoms and are at risk for multiple viral infections, especially in pediatric patients, with a high frequency of mixed infections.^15^, ^16^ Multiplex assays that detect multiple viral gene targets simultaneously in a single tube can have the advantage of rapidly detecting several potential viral pathogens. Multiplex assays offer higher throughput, less detection time, and require less sample volume compared to single‐assay multiplex assays.^17^, ^18^, ^19^ Therefore, rapid and sensitive multiplex detection of respiratory pathogens is important for virus prevention, control, and early treatment.^20^ However, the current conventional nucleic acid detection methods suffer from time‐consuming and insufficient sensitivity to meet the demand for pathogen detection. Previously, we have established a successful single‐plex nested PCR technique combining qPCR and RAA techniques (RAP) with the advantages of ultra‐high sensitivity and rapid detection capability.^11^ However, RAP has not been validated for multiplex pathogen detection of both DNA and RNA viruses (require reverse transcription).

In this study, multiplex RT‐RAP was designed to be run in a single closed‐tube system in less than 1 h. Furthermore, we demonstrated the multiplex detection capability of RAP using HADV3, HADV7, and HRSV. We use a docosane to physically block the RT‐RAA mixes from the qPCR mixes in multiplex RT‐RAP to make the operation more convenient and stable. In addition, multiplex RT‐RAP incorporates an internal reference gene. The internal reference target is an important indicator in the field of nucleic acid detection and is widely used in qPCR and some isothermal nucleic acid detection methods, which can monitor the whole process of sampling, nucleic acid extraction and amplification, effectively reducing false negative results and thus increasing the authenticity of the test results.^21^, ^22^, ^23^

Multiplex RT‐RAP showed high sensitivity and specificity, the detection sensitivity of multiplex RT‐RAP for HADV3, HADV7, and HRSV was 18, 3, and 18 copies per reaction, respectively, which were higher than the corresponding single qPCR/RT‐qPCR reactions and consistent with the detection sensitivity of the corresponding single RAA/RT‐RAA assays. The results of the multiplex RT‐RAP assay for 252 clinical samples were consistent with the results of RT‐qPCR and RT‐RAA. No cross‐reactivity was observed with other common respiratory virus‐positive samples. Test results using nine (each three) serially diluted clinical samples also indicated that multiplex RT‐RAP possessed higher sensitivity than that of correspond qPCR or RT‐qPCR assay. The rapidity of multiplex RT‐RAP (1 h single‐tube analysis) saves running time compared to qPCR/RT‐qPCR. Multiplex RT‐RAP can be performed in a common fluorescence qPCR instrument, and does not require the purchase of a special supporting instrument for versatility.

So far, multiplex RT‐RAP has been applied in our laboratory in the detection of various pathogens including HIV, Norovirus GII, Rotavirus, and SNP detection of Mycobacterium tuberculosis. Our preliminary results indicate multiplex RT‐RAP is a universal sensitive and rapid nucleic acid detection technique. Although, we have made various improvements over the previous single‐plex RAP technique, which has greatly improved the operation and stability of the method, the components of the RAA system are not yet compatible with those of PCR, for example, the RAA mixes still need to physically block the two mixes, which cannot really achieve a single mix throughout the detection process. In addition, there is no obvious correlation between RAA enrichment and the amount of initial viral nucleic acid, the quantitative detection of RAP method still needs further research. This method is not sufficiently automated or mechanized. Our ongoing attempt is to use microfluidic chip technology to improve the automation and the throughput of the assay.

Despite the current multiplex RAP technology still has some shortcomings, it remains a robust, rapid, and sensitive nucleic acid detection technique that can be applied to viral, bacterial, and mutant loci detection. In addition, due to its high sensitivity, it might be useful for rapid screening of asymptomatic patients with infections, sensitive detection of cerebrospinal fluid samples with low viral load, and low frequency monitoring of drug‐resistant pathogens.

In conclusion, the proposed closed‐tube multiplex RT‐RAP is a rapid, highly specific, and highly sensitive nucleic acid detection platform, which has great potential to be used in the detection of various pathogens.

## AUTHOR CONTRIBUTIONS

XM and XS designed the study. GF, RZ, FT, XS, MZ, and FT performed the experiments. GF, RZ, and XH analyzed and interpreted the data. XM and GF wrote the study. All authors provided a critical review and approved the final article.

## FUNDING INFORMATION

This work was supported by grants from the National Key R&D Program of China (2021YFC2301102), National Natural Science Foundation of China (82202593), Natural Science Foundation of Shandong Province, China (ZR2022MH115), and the key R&D projects in Zibo city (2020kj100011).

## CONFLICT OF INTEREST STATEMENT

The authors declare that there is no conflict of interest in this work.

## Data Availability

The data that support the findings of this study are available from the corresponding author upon reasonable request.
